# The Fly as a Carrier of Tuberculosis Infection

**Published:** 1904-11

**Authors:** 


### THE FLY AS A CARRIER OF TUBERCULOSIS INFECTION.*

The fact that bacteria are the etiological factor of the majority of the most fatal diseases is now well established, and the efforts of the sanitarian are turned towards determining the avenues of transmission and methods of combating the spread of these infections. The writer, while investigating the possibility of the spread of infection through the feces of flies feeding on the excreta of typhoid patients, and noticing the number of flies that flock around the cuspidors of those suffering from pulmonary tuberculosis, was led to a series of experiments in this connection. The fly used in these experiments was the common house fly (*Musca domestica*) and the blue bottle (*Musca cæsar*). In each experiment fourteen flies were used, which were kept confined twenty-four hours previously in cages made of wire screen, and fed on milk; clean cover glasses were introduced into the cages and on these they willingly defecated, after which the covers were stained by the Ziehl-Nielsons method to determine if tubercle bacilli were present or not. The results were negative.

The first experiment was made on flies caught feeding on the bottles containing tuberculous sputum that came into the laboratory for examination. These were supplied with clean cover slips which they speedily soiled. Sixteen of the covers, ten of which contained tubercle bacilli, were stained and examined. The next two batches of flies were fed on tuberculosis sputum in which the bacilli had previously been demonstrated. The sputum was placed on watch glasses and covered with a fine wire screen. On this screen the fly could walk without getting its feet or wings in the sputum and could feed through the meshes. The flies were also fed on milk, but they seemed to prefer the sputum. Six hours after feeding, clean cover glasses were introduced and left from three to four hours, during which time considerable feces were deposited on them. They were then withdrawn and stained, and all showed tubercle bacilli. The flies apparently suffered from diarrhea after feeding on the sputum, as they defecated more after the infectious material was ingested than before.

*Read by E. H. Hayward, M.D., Detroit, Mich., Bacteriologist to the School Board of Health, before the Detroit Pathological Society.

In the fourth experiment, the same conditions were observed, but the flies were watched and as soon as a cover was soiled, it was withdrawn and examined with the same results as the preceding experiment. The flies all died from two to three days after the infectious material was introduced, although the controls fed on milk lived from eight to ten days in confinement.

Culture plates made of feces on glycerine agar and incubated two weeks showed a growth of tubercle bacilli, thus proving that the vitality of the tubercle bacillus is not impaired by passage through the intestinal canal of the fly. Post-mortems were made from each batch and smears from the stomach and intestines showed large numbers of tubercle bacilli.

The feces were also rubbed up with sterile water and injected into the peritoneal cavity of guinea-pigs, which developed tuberculosis.

In view of the omnipresence of the house-fly and the indiscriminate manner in which it deposits its feces, the opportunity for the spread of infection is manifest, particularly when it is taken into consideration that this material is charged with all varieties of pathogenic bacteria. The fact that the tubercle bacillus may be transported from the focus of infection in the lung of one person to the alimentary tract of another in the course of a few hours is highly suggestive of a common mode of transmission. Although the bacilli pass through the alimentary canal of the fly, their virulence is apparently unaltered and, therefore, we must consider such infections almost direct.

This report is a preliminary one, to be followed by others with more exhaustive experiments.—*New York Medical Journal and Philadelphia Medical Journal.*

